# Adjuvant Radiotherapy Is Associated with an Increase in the Survival of Old (Aged over 80 Years) and Very Old (Aged over 90 Years) Women with Breast Cancer Receiving Breast-Conserving Surgery

**DOI:** 10.3390/jpm12020287

**Published:** 2022-02-16

**Authors:** Chung-Chien Huang, Chia-Lun Chang, Mingyang Sun, Ming-Feng Chiang, Shao-Yin Sum, Jiaqiang Zhang, Szu-Yuan Wu

**Affiliations:** 1 International Ph.D. Program in Biotech and Healthcare Management, School of Health Care Administration, College of Management, Taipei Medical University, Taipei 110, Taiwan; cc-test@tmu.edu.tw; 2 Department of Medical Quality, Taipei Municipal Wan Fang Hospital, Taipei Medical University, Taipei 110, Taiwan; 3 Department of Hemato-Oncology, Wan Fang Hospital, Taipei Medical University, Taipei 110, Taiwan; richardch9@hotmail.com; 4 Department of Internal Medicine, School of Medicine, College of Medicine, Taipei Medical University, Taipei 110, Taiwan; 5 Department of Anesthesiology and Perioperative Medicine, Henan Provincial People’s Hospital, People’s Hospital of Zhengzhou University, Zhengzhou 450052, China; mingyangsun1986@163.com (M.S.); jiaqiang197628@163.com (J.Z.); 6 Division of Gastroenterology and Hepatology, Department of Internal Medicine, Lo-Hsu Medical Foundation, Lotung Poh-Ai Hospital, Yilan 265, Taiwan; chiangmingf@gmail.com; 7 Department of General Surgery, Lo-Hsu Medical Foundation, Lotung Poh-Ai Hospital, Yilan 265, Taiwan; b91401126@ntu.edu.tw; 8 Department of Food Nutrition and Health Biotechnology, College of Medical and Health Science, Asia University, Taichung 413, Taiwan; 9 Big Data Center, Lo-Hsu Medical Foundation, Lotung Poh-Ai Hospital, Yilan 265, Taiwan; 10 Graduate Institute of Business Administration, College of Management, Fu Jen Catholic University, Taipei 242062, Taiwan; 11 Division of Radiation Oncology, Lo-Hsu Medical Foundation, Lotung Poh-Ai Hospital, Yilan 265, Taiwan; 12 Department of Healthcare Administration, College of Medical and Health Science, Asia University, Taichung 413, Taiwan; 13 Cancer Center, Lo-Hsu Medical Foundation, Lotung Poh-Ai Hospital, Yilan 265, Taiwan; 14 Centers for Regional Anesthesia and Pain Medicine, Taipei Municipal Wan Fang Hospital, Taipei Medical University, Taipei 110, Taiwan

**Keywords:** breast cancer, old age, breast-conserving surgery, radiotherapy, survival

## Abstract

This study is the first to examine the effect of adjuvant whole-breast radiotherapy (WBRT) on oncologic outcomes such as all-cause death, locoregional recurrence (LRR), and distant metastasis (DM) in old (aged ≥80 years) and very old (aged ≥90 years) women with breast invasive ductal carcinoma (IDC) receiving breast-conserving surgery. After propensity score matching, adjuvant WBRT was associated with decreases in all-cause death, LRR, and DM in old and very old women with IDC compared with no use of adjuvant WBRT. **Background**: To date, no data on the effect of adjuvant whole-breast radiotherapy (WBRT) on oncologic outcomes, such as all-cause death, locoregional recurrence (LRR), and distant metastasis (DM), are available for old (aged ≥80 years) and very old (≥90 years) women with breast invasive ductal carcinoma (IDC) receiving breast-conserving conservative surgery (BCS). **Patients and Methods**: We enrolled old (≥80 years old) and very old (≥90 years old) women with breast IDC who had received BCS followed by adjuvant WBRT or no adjuvant WBRT. We grouped them based on adjuvant WBRT status and compared their overall survival (OS), LRR, and DM outcomes. To reduce the effects of potential confounders when comparing all-cause mortality between the groups, propensity score matching was performed. **Results**: Overall, 752 older women with IDC received BCS followed by adjuvant WBRT, and 752 with IDC received BCS with no adjuvant WBRT. In multivariable Cox regression analysis, the adjusted hazard ratio (aHR) and 95% confidence interval (95% CI) of all-cause death for adjuvant WBRT compared with no adjuvant WBRT in older women with IDC receiving BCS was 0.56 (0.44–0.70). The aHRs (95% CIs) of LRR and DM for adjuvant WBRT were 0.29 (0.19–0.45) and 0.45 (0.32–0.62), respectively, compared with no adjuvant WBRT. **Conclusions**: Adjuvant WBRT was associated with decreases in all-cause death, LRR, and DM in old (aged ≥80 years) and very old (aged ≥90 years) women with IDC compared with no adjuvant WBRT.

## 1. Introduction

Standard treatments based on cancer treatment guidelines such as the National Comprehensive Cancer Network (NCCN) guidelines are not suitable for every older patient, because many randomized controlled trials (RCTs) for breast cancer therapy do not enroll patients ≥65 years old [1]. Determining optimal treatments for older cancer patients is challenging, especially for those aged 80 years or more. Although trials have enrolled patients ≥70 years old, the sample size of those ≥80 years old is small, and trials including those ≥90 years old are scant [2,3]. However, cancer is commonly a disease of the old, and the median age at diagnosis for all sites is 65 years [4]. Older patients (≥80 years) constitute a substantial percentage of those with breast cancer [5]. Approximately one in four patients with breast cancer are aged more than 65 years, and approximately 10% of the total breast cancer population is 80 years or older [5]. This age group often presents challenges in terms of treatment because of comorbidities and frailty [6].

It is difficult to evaluate long-time overall survival and disease-free survival for elderly breast cancer patients in RCTs, due to their short life-expectancy. Additionally, there is also the cost of treatment to consider in elderly patients with short life-expectancies. Therefore, all comorbidities should be considered in these kinds of elderly patient studies, and be well-matched through propensity score matching (PSM). Most patients should have Charlson comorbidity index (CCI) score of 0–1 with relative health, which might be an association with longer life-expectancy. The selection of relatively healthy, suitable elderly breast cancer patients for the consideration of further adjuvant radiotherapy (RT) would be worthwhile. 

Adjuvant RT is applied to eradicate any tumor deposits remaining following surgery [7]. This reduces the risk of locoregional recurrence (LRR) and improves breast cancer-specific survival and overall survival (OS) [7]. For most women treated with breast-conserving surgery (BCS), adjuvant whole-breast RT (WBRT), rather than surgery alone, is recommended according to the NCCN guidelines and the results of RCTs [1,7]. Studies with grade 2B evidence (weak recommendation) have suggested that the omission of RT might be considered in women ≥65 years old with node-negative, hormone receptor-positive, human epidermal growth factor receptor 2 (HER2)-negative primary tumors up to 3 cm, for whom endocrine therapy is planned [8,9,10,11,12]; alternatively, administering RT to these women is also reasonable depending on their values and preferences, and the biologic features of the tumor. For example, women in this subset who wish to minimize their risk of LRR and accept the toxicities associated with RT may reasonably opt for RT. To date, no study with a sufficient sample size and long-term follow-up for older (≥80 years old) women with breast cancer has been conducted; this is especially true for 90-year-old women and above. A head-to-head PSM study mimicking an RCT might be necessary, especially for old (≥80 years) and very old (≥90 years) women.

The radiation oncologist should discuss the advantages and disadvantages of RT with older women with breast cancer receiving BCS prior to making a decision on its omission. For example, in the real world, compliance with endocrine therapy is a critical aspect of treatment, particularly for those with RT omission. A head-to-head study with a sufficiently large sample size and long follow-up is required to estimate the oncologic outcomes of adjuvant WBRT for older women with breast cancer undergoing BCS. We conducted this PSM study to examine the effects of adjuvant WBRT on oncologic outcomes such as OS, LRR, and distant metastasis (DM) in old (aged ≥80 years) and very old (aged ≥90 years) women, who have scarcely been enrolled in RCTs; these findings would help determine the value of adjuvant WBRT in these patients.

## 2. Patients and Methods

### 2.1. Study Population

In this cohort study, data were retrieved from the Taiwan Cancer Registry Database (TCRD). We enrolled old (age ≥80 years) and very old (≥90 years) women with breast invasive ductal carcinoma (IDC) who had received BCS between 1 January 2008 and 31 December 2018. The index date was the date of BCS, and the follow-up duration was from the index date to 31 December 2019. The TCRD of the Collaboration Center of Health Information Application contains detailed cancer-related information of patients, including clinical stage, pathologic stage, chemotherapy regimen, chemotherapy dose, molecular status, drug use, hormone receptor status, HER2 status, radiation modality and dose, and surgical procedure [13,14,15,16]. The study protocols were reviewed and approved by the Institutional Review Board of Tzu-Chi Medical Foundation (IRB109-015-B).

### 2.2. Inclusion and Exclusion Criteria

The diagnoses of the enrolled women with breast IDC were confirmed after their pathological data were reviewed, and women with newly diagnosed IDC were confirmed to have no other cancers or DMs. Women with IDC were included if they were 80 years or older and had clinical stage IA-IIIC (American Joint Committee on Cancer [AJCC], 8th edition) without metastasis. Women with IDC were excluded if they had a history of cancer before the IDC diagnosis date, unknown pathologic types, missing sex data, unclear staging, or non-IDC histology. In addition, women having unclear differentiation of the tumor grade, missing data on hormone receptor status, or unknown HER2 status were excluded. Other adjuvant treatments such as chemotherapy, hormone therapy, or HER2 inhibitors did not constitute exclusion criteria based on the NCCN guidelines [17]. We also excluded women with unclear data on surgical procedures such as BCS or TM, ill-defined nodal surgery, or unclear Charlson comorbidity index (CCI) scores. Hormone receptor-positivity was defined as ≥1% of tumor cells demonstrating positive nuclear staining through immunohistochemistry [18].

After applying the inclusion and exclusion criteria, we divided the population into two groups based on their adjuvant WBRT status to compare all-cause mortality: Group 1 (older women with IDC who received BCS followed by adjuvant WBRT) and Group 2 (older women with IDC who received BCS and no adjuvant WBRT). We also excluded women in Group 1 receiving nonstandard adjuvant WBRT (contrast with standard adjuvant radiotherapy consisting of irradiation to the whole breast with a minimum of 50 Gy). Contemporary RT techniques (i.e., three-dimensional RT and intensity-modulated RT) were included, and the conventional two-dimensional RT technique was excluded. The incidence of comorbidities was scored using the CCI [19,20]. Only comorbidities observed within 6 months before the index date were included; they were classified according to International Classification of Diseases, 10th Revision, Clinical Modification codes (ICD-10-CM codes) at the first admission or based on more than two repetitions of a code recorded at outpatient department visits.

### 2.3. Study Covariates and Propensity Score Matching

To reduce the effects of potential confounders when comparing all-cause mortality between the adjuvant WBRT and nonadjuvant WBRT groups, PSM was performed. A greedy method was used to match the cohorts at a 1:1 ratio by age, tumor differentiation, AJCC clinical stage, AJCC pathologic stage, pT, pN, neoadjuvant chemotherapy, adjuvant chemotherapy, hormone receptor status, HER2 status, nodal surgical type, CCI score, hypertension, ischemic heart disease, cerebrovascular disease, chronic obstructive pulmonary disease (COPD), diabetes, hospital level (medical center or not), hospital region, and income with a propensity score within a caliper of 0.2 [21]. Moreover, we separated covariates such as hypertension, ischemic heart disease, cerebrovascular disease, COPD, and diabetes [22] from CCI scores and considered these covariates independently in PSM for more precise matching to control for confounders of all-cause death.

### 2.4. Statistics

Multivariable Cox regression analysis was performed to calculate hazard ratios (HRs) to determine the potential independent predictors of all-cause death, LRR, and DM. PSM was applied to control for potential predictors in the analysis (Table 1), and all-cause death was the primary endpoint in the two groups. LRR and DM were secondary endpoints and were estimated using proportional subdistribution hazard regression to overcome the competing risk of death in the analysis of time-to-event data [23,24].

The cumulative incidence of death was estimated using the Kaplan–Meier method, and differences in OS, LRR-free survival, and DM-free survival between older women receiving BCS followed by adjuvant WBRT and those without adjuvant WBRT were determined using a log-rank test. We performed all analyses using SAS version 9.3 (SAS Institute, Cary, NC, USA). *p* values < 0.05 were considered statistically significant in the two-tailed Wald test. Risk of all-cause death was calculated, and subgroup analyses by age and cancer were conducted using a log-rank test.

## 3. Results

### 3.1. Study Cohort

After PSM, 1504 older women with balanced covariates were included (Table 1). Among them, 752 received BCS followed by adjuvant WBRT (Group 1) and 752 with IDC received BCS without adjuvant WBRT (Group 2). After PSM, the results revealed that the covariates between the groups were homogenous. The median follow-up durations after the index date were 70.3 and 64.4 months for Group 1 and Group 2, respectively.

### 3.2. Impact of Adjuvant WBRT on Oncologic Outcomes of Old and Very Old Women

In multivariable Cox regression analysis, the adjusted HR (aHR) and 95% confidence interval (95% CI) of all-cause death for adjuvant WBRT compared with no adjuvant WBRT was 0.56 (0.44–0.70). The aHRs (95% CIs) of LRR and DM for adjuvant WBRT were 0.29 (0.19–0.45) and 0.45 (0.32–0.62), respectively, compared with no adjuvant WBRT. The aHRs (95% CIs) of all-cause death for old age (85–89 years) and very old age (≥90 years) were 1.85 (1.28–2.69) and 1.67 (1.47–3.46), respectively, compared with the age of 80–84 years. Other confounders were not significantly different for all-cause death, LRR, and DM between the two groups because of the well-matched PSM design without residual imbalance [25,26].

### 3.3. Age Stratification in Multivariable Cox Regression Analysis

Because age remained an independent prognostic factor of all-cause death even after PSM, residual imbalance existed in the confounder of age for all-cause death (Table 2). We performed multivariable analysis of OS that was stratified by the ages of 80–89 years and ≥90 years (Table 3). The aHRs (95% CIs) of all-cause mortality for adjuvant WBRT compared with no adjuvant WBRT in old (80–89 years) and very old (≥90 years) women receiving BCS were 0.60 (0.40–0.91) and 0.64 (0.48–0.87), respectively (Table 3). In addition, the aHR (95% CI) of all-cause death for the age of 85–89 was 1.48 (1.07–2.27), compared with the age of 80–84 years, and that for the age of ≥95 years was 1.50 (1.10–2.04) compared with the age of 90–94 years.

### 3.4. Survival Curves with or without Adjuvant WBRT

**Figure 1:**
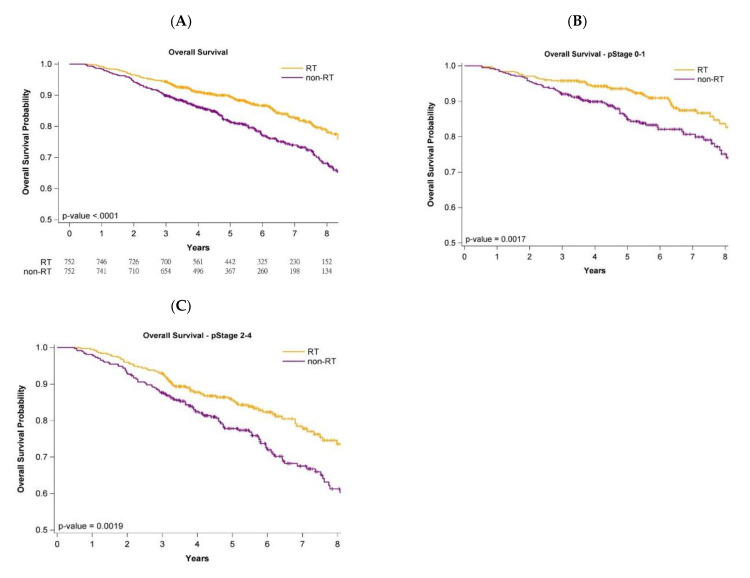
KM curves for overall survival after propensity score matching in patients aged ≥80 years undergoing breast conservative surgery. (**A**)—All stages; (**B**)—Stage 0–1; (**C**)—Stage 2–4.

**Figure 2:**
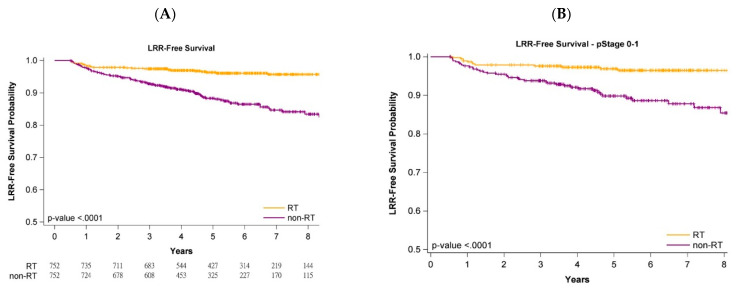

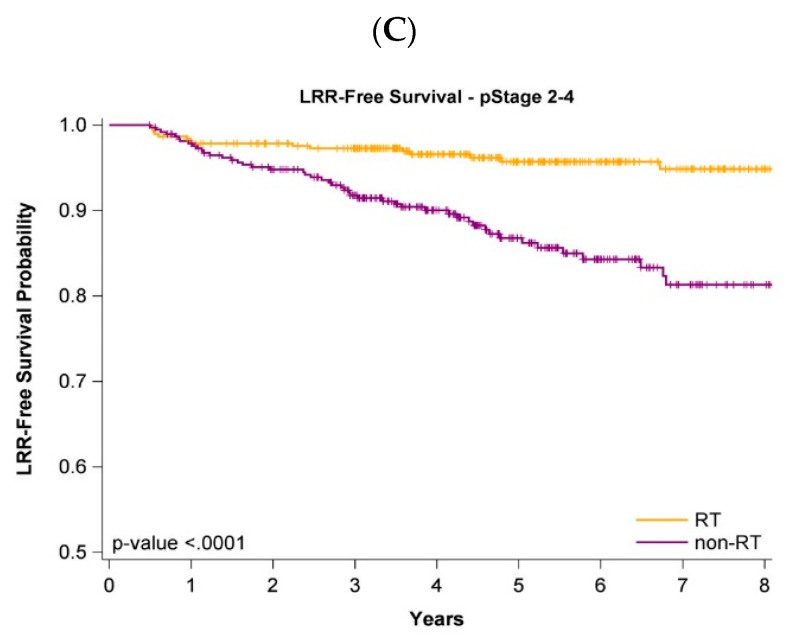
KM survival curves for local recurrence after propensity score matching in patients aged ≥80 years undergoing breast conservative surgery. (**A**)—All stages; (**B**)—Stage 0–1; (**C**)—Stage 2–4.

**Figure 3:**
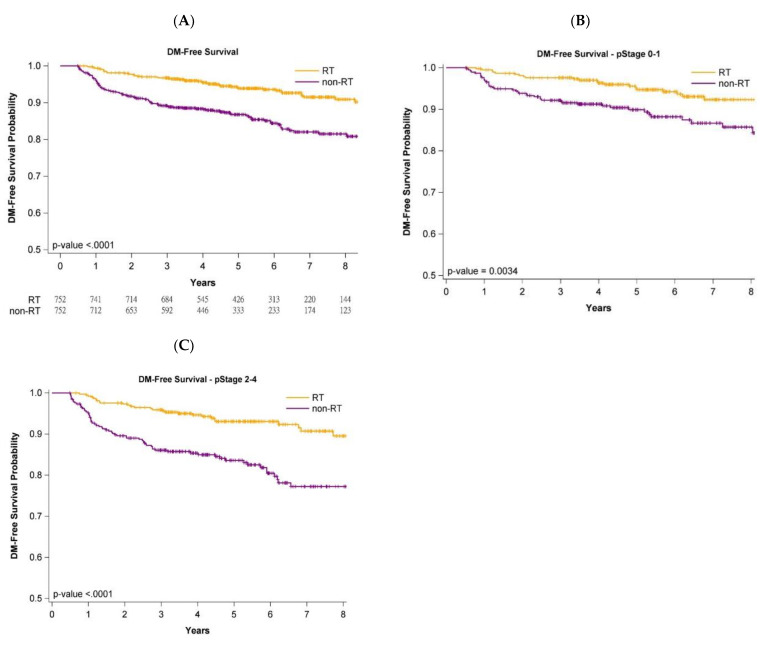
KM survival curves for distant metastasis after propensity score matching in patients aged ≥80 years undergoing breast conservative surgery. (**A**)—All stages; (**B**)—Stage 0–1; (**C**)—Stage 2–4.

Figure 1, Figure 2 and Figure 3 present Kaplan–Meier curves that illustrate the overall, LRR-free, and DM-free survival curves of the groups. The 5-year OS probability was 90.11% and 83.92% in the adjuvant WBRT and nonadjuvant WBRT groups, respectively (Figure 1A) (log-rank test, *p* < 0.0001). Additionally, 5-year LRR-free survival was 97.81% and 87.32% in the adjuvant WBRT group and nonadjuvant WBRT group, respectively (Figure 2A; log-rank test, *p* < 0.0001). Moreover, 5-year DM-free survival was 95.74% and 85.61% in the adjuvant WBRT group and nonadjuvant WBRT group, respectively (Figure 3A; log-rank test, *p* < 0.0001).

### 3.5. Survival Curves of Cancer Stages and Age Stratification

Analysis of the impact of stage (early stage (stage 0-I) or advanced stage (stage II-III)) on oncologic outcomes (OS, LRR, and DM) was conducted with stratification by pathologic stages. The OS, LRR-free, and DM-free survival curves of the adjuvant WBRT group remained significantly superior to those of the nonadjuvant WBRT group regardless of stage (Figure 1B,C, Figure 2B,C and Figure 3B,C). Age stratification by 80–89 and ≥90 years was also performed. The OS, LRR-free, and DM-free survival curves of the adjuvant WBRT group were significantly superior to those of the non-adjuvant WBRT group in both stratifications (Supplementary Figures S1 and S2).

## 4. Discussion

### 4.1. No Solution Regarding Adjuvant WBRT for Older Women with Breast Cancer

Breast cancer is the most common cancer in women, and one in ten patients affected are aged ≥80 years [5]. However, this age group is generally excluded from clinical trials, and data to inform their care are sparse [7]. Additionally, no RCT with women aged ≥90 years with breast cancer has been conducted. In practice, treatment for older patients with breast cancer involves shared decision-making between physicians and patients based on expected survival lifespan, comorbidities, or prognostic factors of tumor recurrence [8,9,10,11,12]. Nevertheless, few patients in the ≥80 years age group receive RT as part of their treatment, especially those aged ≥90 years [5,8,9,10,11,12,27]. Studies on the omission of RT in older women with a low recurrence of hormone receptor-positive or HER2-negative breast cancer (as a better prognosis) have been conducted, but studies including women aged ≥80 years are scant [8,9,10,11,12]. Breast cancer biologic subtypes of women aged ≥80 years exhibit similarities with those of younger postmenopausal women; thus, treatments should be consistent [6]. Possible problems are the expected survival and comorbidities contributing to the incidence of LRR- and DM-related mortality [22,23,24]. Nonetheless, if older patients with IDC receiving BCS have consistent comorbidities, molecular types (similar hormone receptor status and HER2), the same cancer stages, and similar treatment protocols relative to younger patients, whether adjuvant WBRT should be omitted is unclear.

### 4.2. Value of PSM in This Population

As shown in Table 1, all potential cofounders of all-cause death for women with breast cancer were matched and controlled through PSM. The cofounders—age, differentiation, AJCC clinical stage, AJCC pathologic stage, pT, pN, neoadjuvant chemotherapy, adjuvant chemotherapy, hormone receptor status, HER2 status, nodal surgical type, CCI score, hospital level (medical center or not), hospital region, and income, all mentioned in previous studies—were matched to balance covariates between the two groups [13,14,15,28,29,30,31]. Because the most common causes of death in older patients are hypertension, ischemic heart disease, cerebrovascular disease, COPD, and diabetes [22], we separated the covariates from the CCI scores and included these covariates in PSM independently for more precise matching to control the confounders of all-cause mortality. PSM allows the design of an observational (non-randomized) study that mimics some of the characteristics of an RCT [32]. After PSM design, we believe the balanced covariates mimic an RCT [32] in our study without selection bias for adjuvant WBRT and no adjuvant WBRT in older women receiving BCS. Before PSM, the trends of selection of no adjuvant WBRT (raw population in Table 1) were compatible with those in previous studies, in which women with node-negative, hormone receptor-positive, HER2-negative cancer or small tumor sizes preferred no adjuvant RT [8,9,10,11,12]. Our findings indicate that women with favorable prognostic factors of OS would not receive adjuvant WBRT (Table 1). Conducting an RCT with patients ≥80 years old is difficult. Therefore, a PSM study with balanced conditions is appropriate for evaluating the value of adjuvant WBRT for older women.

### 4.3. Conditions Different from Previous Studies

Adjuvant WBRT can be omitted in older (≥65 years) women with hormone receptor-positive breast cancer, especially for clinically node-negative, small, or HER2-negative breast cancer [8,9,10,11,12]. Moreover, omission of RT in patients with hormone receptor-positive, node-negative, small breast cancer is supported by a meta-analysis that included postmenopausal women, all of whom received systemic therapy (the majority received tamoxifen) [3]. However, most women had T1, node-negative tumors and were aged ≥65 years, with 39% aged ≥70 years [3]. Only approximately 10% of patients were ≥80 years old in the aforementioned studies [3,8,9,10,11,12]. Comorbidities were not considered in the previous studies with unexpected survival lifespans [3,8,9,10,11,12], and the survival benefit of adjuvant WBRT could not be determined in the aforementioned reports. In the current study, all the enrolled women were ≥80 years old, and approximately 40% were ≥90 years old (Table 1). All comorbidities were considered in our study and were well-matched through PSM. In addition, molecular type, cancer stage, and treatment protocols were controlled for through PSM. Therefore, our study is the first head-to-head PSM study mimicking an RCT with consistent conditions to estimate the oncologic outcomes after adjuvant WBRT in old (aged 80–89 years) and very old (aged ≥90 years) women with IDC receiving BCS.

### 4.4. Cancer Stage and Age Stratification

Because some reports have indicated that adjuvant RT can be omitted in older women with early-stage breast cancer receiving mastectomy [2,10], we estimated the effects of adjuvant WBRT by using the log-rank test for the PSM population stratified by early or advanced pathologic stage. The results indicated receiving that adjuvant WBRT was significantly superior to not receiving adjuvant WBRT for OS, LRR-free survival, and DM-free survival, even in the earliest stages (stage 0-I) (Figure 1B, Figure 2B and Figure 3B). Previous studies reporting no significant survival difference between adjuvant RT and no adjuvant RT for breast cancer in older women might be attributed to small sample size, short follow-up time, or unknown comorbidities [2,10]. The most common cause of death in these older women is comorbidities [22], but no data on comorbidities have been included in reports [2,10]. Another key concern is that those aged ≥80 years were not the main population, and that those aged ≥90 years were few in the aforementioned studies [2,10]. We used the log-rank test for investigating the effect of adjuvant WBRT or no adjuvant WBRT on oncologic outcomes for different age groups (80–89 years and ≥90 years) in the PSM population (Supplementary Figures S1 and S2). No study of patients aged ≥90 years with breast cancer has been conducted. Our study is the first to demonstrate the benefits of adjuvant RT for women 90 years or older with IDC receiving BCS.

### 4.5. Limitations

This study has limitations. First, because all the women with IDC were enrolled from an Asian population, the corresponding ethnic susceptibility compared with non-Asian populations remains unclear; hence, our results should be cautiously extrapolated to non-Asian populations. However, no evidence suggests differences in oncologic outcomes between Asian and non-Asian women with breast IDC receiving BCS. Second, the diagnoses of all comorbid conditions were based on ICD-10-CM codes. However, the combination of the TCRD and the National Health Insurance Research Database (NHIRD) in Taiwan appears to be a valid resource for population research on cardiovascular diseases, stroke, or chronic comorbidities [33,34,35]. The Taiwan Cancer Registry Administration randomly reviews charts and interviews patients to verify the accuracy of diagnoses, and hospitals with outlier chargers or practices may be audited and heavily penalized if malpractice or discrepancies are identified. Accordingly, to obtain crucial information on population specificity and disease occurrence, a large-scale RCT comparing carefully selected patients undergoing suitable treatments is essential. However, as mentioned, enrolling patients ≥80 or even ≥90 years of age in an RCT is difficult. Despite its limitations, a major strength of this study is the use of a nationwide population-based registry with detailed baseline and treatment information. Lifelong follow-up was possible through the linkage of the registry with the national Cause of Death database. Considering the magnitude and statistical significance of the observed effects in the current study, the limitations are unlikely to affect our conclusions.

## 5. Conclusions

Compared with no adjuvant WBRT, adjuvant WBRT may be associated with decreased all-cause of death, LRR, and DM for older women with breast IDC receiving BCS regardless of stage (early vs. advanced) and age (80–89 vs. ≥90 years). We suggest adjuvant WBRT for old or very old women with IDC receiving BCS, even if the cancer stage is early or the patient is 90 years or older.

## Figures and Tables

**Table 1:** Demographic information of patients aged ≥80 years undergoing breast conservative surgery.

		Raw Population							Propensity Score-Matched Population				
		Total N = 3703		Adjuvant WBRT N = 2776		Non-WBRT N = 927			Adjuvant WBRT N = 752		Non-WBRT N = 752		
Variables		*n*	(%)	*n*	(%)	*n*	(%)	*p* Value	*n*	(%)	*n*	(%)	*p* Value
Age	Mean (SD)	84.8	(6.1)	84.4	(4.9)	85.9	(7.2)	<0.0001	85.3	(6.0)	85.9	(6.3)	0.9674
	Median (IQR, Q1–Q3)	84	(81–88)	84	(81–88)	84	(82–89)		84	(82–89)	84	(82–90)	
	80–84	1815	(49.0)	1598	(57.6)	217	(23.4)	<0.0001	215	(28.6)	215	(28.6)	1.0000
	85–89	1285	(34.7)	879	(31.7)	406	(43.8)		238	(31.6)	238	(31.6)	
	90+	603	(16.3)	299	(10.8)	304	(32.8)		299	(39.8)	299	(39.8)	
Differentiation	I	851	(23.0)	631	(22.7)	220	(23.7)	0.3441	171	(22.7)	182	(24.2)	0.3075
	II	2071	(55.9)	1544	(55.6)	527	(56.9)		406	(54.0)	418	(55.6)	
	III	781	(21.1)	601	(21.6)	180	(19.4)		175	(23.3)	152	(20.2)	
AJCC Clinical stage	I	2033	(54.9)	1568	(56.5)	465	(50.2)	0.0012	402	(53.5)	398	(52.9)	0.9532
	II	1547	(41.8)	1112	(40.1)	435	(46.9)		329	(43.8)	332	(44.1)	
	III	123	(3.3)	96	(3.5)	27	(2.9)		21	(2.8)	22	(2.9)	
AJCC Pathologic stage	0	41	(1.1)	33	(1.2)	8	(0.9)	0.0029	4	(0.5)	4	(0.5)	1.0000
	I	1894	(51.1)	1449	(52.2)	445	(48.0)		375	(49.9)	375	(49.9)	
	II	1531	(41.3)	1103	(39.7)	428	(46.2)		331	(44.0)	331	(44.0)	
	III	237	(6.4)	191	(6.9)	46	(5.0)		42	(5.6)	42	(5.6)	
pT	0	58	(1.6)	47	(1.7)	11	(1.2)	<0.0001	5	(0.7)	6	(0.8)	0.5977
	1	2214	(59.8)	1710	(61.6)	504	(54.4)		429	(57.0)	424	(56.4)	
	2	1356	(36.6)	975	(35.1)	381	(41.1)		301	(40.0)	301	(40.0)	
	3	45	(1.2)	24	(0.9)	21	(2.3)		8	(1.1)	14	(1.9)	
	4	30	(0.8)	20	(0.7)	10	(1.1)		9	(1.2)	7	(0.9)	
pT	0–1	2272	(61.4)	1757	(63.3)	515	(55.6)	<0.0001	434	(57.7)	430	(57.2)	0.6625
	2–4	1431	(38.6)	1019	(36.7)	412	(44.4)		318	(42.3)	322	(42.8)	
pN	0	2890	(78.0)	2122	(76.4)	768	(82.8)	0.0004	618	(82.2)	608	(80.9)	0.8552
	1	613	(16.6)	488	(17.6)	125	(13.5)		103	(13.7)	113	(15.0)	
	2	140	(3.8)	114	(4.1)	26	(2.8)		22	(2.9)	23	(3.1)	
	3	60	(1.6)	52	(1.9)	8	(0.9)		9	(1.2)	8	(1.1)	
pN	0	2890	(78.0)	2122	(76.4)	768	(82.8)	<0.0001	618	(82.2)	608	(80.9)	0.4111
	1+	813	(22.0)	654	(23.6)	159	(17.2)		134	(17.8)	144	(19.1)	
Neoadjuvant Chemotherapy		115	(3.1)	107	(3.9)	8	(0.9)	<0.0001	8	(1.1)	7	(0.9)	0.7389
Adjuvant chemotherapy		1270	(34.3)	1126	(40.6)	144	(15.5)	<0.0001	162	(21.5)	142	(18.9)	0.0588
Hormone receptor positive		1871	(50.5)	1394	(50.2)	477	(51.5)	0.5132	368	(48.9)	372	(49.5)	0.8168
HER2 positive		231	(6.2)	189	(6.8)	42	(4.5)	0.0130	41	(5.5)	40	(5.3)	0.9081
Nodal surgery	ALND	2259	(61.0)	1688	(60.8)	571	(61.6)	0.6301	432	(57.4)	424	(56.4)	0.3608
	SLNB	1444	(39.0)	1088	(39.2)	356	(38.4)		320	(42.6)	328	(43.6)	
CCI Scores	0	1513	(40.9)	1178	(42.4)	335	(36.1)	<0.0001	279	(37.1)	283	(37.6)	0.9752
	1	1133	(30.6)	863	(31.1)	270	(29.1)		226	(30.1)	224	(29.8)	
	2+	1057	(28.5)	735	(26.5)	322	(34.7)		247	(32.8)	245	(32.6)	
Hypertension		2430	(65.6)	1765	(63.6)	665	(71.7)	<0.0001	543	(72.2)	530	(70.5)	0.4460
Ischemic heart diseases		925	(25.0)	582	(21.0)	343	(37.0)	<0.0001	260	(34.6)	258	(34.3)	0.9811
Cerebrovascular diseases		377	(10.2)	229	(8.2)	148	(17.0)	<0.0001	125	(16.6)	117	(15.6)	0.2624
COPD		552	(14.9)	301	(10.8)	251	(27.1)	<0.0001	211	(28.1)	211	(128.1)	1.0000
Diabetes		1180	(31.9)	862	(31.1)	318	(34.3)	0.0658	268	(35.6)	254	(33.8)	0.4423
Hospital level	Medical center	1973	(53.3)	1394	(50.2)	579	(62.5)	<0.0001	446	(59.3)	461	(61.3)	0.3258
	Non-Medical centers	1730	(46.7)	1382	(49.8)	348	(37.5)		306	(40.7)	291	(38.7)	
Hospital area	North	2017	(54.5)	1563	(56.3)	454	(49.0)	<0.0001	384	(51.1)	374	(49.7)	0.5139
	Center	761	(20.6)	489	(17.6)	272	(29.3)		196	(26.1)	211	(28.1)	
	South/East	925	(25.0)	724	(26.1)	201	(21.7)		172	(22.9)	167	(22.2)	
Income	<NTD 18,000	1331	(35.9)	987	(35.6)	344	(37.1)	0.0599	279	(37.1)	281	(37.4)	0.9108
	NTD 18,000–24,000	1240	(33.5)	928	(33.4)	312	(33.7)		240	(31.9)	248	(33.0)	
	NTD 24,000–36,000	350	(9.5)	283	(10.2)	67	(7.2)		55	(7.3)	56	(7.4)	
	NTD 36,000+	782	(21.1)	578	(20.8)	204	(22.0)		178	(23.7)	167	(22.2)	
Follow-up time, months	Mean (SD)	68.8	(29.1)	70.7	(28.7)	63.1	(29.3)		70.3	(29.2)	64.4	(28.8)	
Death		606	(16.4)	336	(12.1)	270	(29.1)	<0.0001	123	(16.4)	182	(24.2)	<0.0001
Locoregional recurrence		245	(6.6)	144	(5.2)	101	(10.9)	<0.0001	28	(3.7)	88	(11.7)	<0.0001
Distant metastasis		331	(8.9)	214	(7.7)	117	(12.6)	<0.0001	54	(7.2)	108	(14.4)	<0.0001

SD—standard deviation; IQR—interquartile range; WBRT—whole breast radiotherapy; AJCC—American Joint Committee on Cancer; HER2—human epidermal growth factor receptor 2; SLNB—sentinel lymph node biopsy; ALND—axillary lymph node dissection; CCI—Charlson comorbidity index; pT—pathologic tumor stage; pN—pathologic nodal stage; COPD—chronic obstructive pulmonary disease.

**Table 2:** Multivariate analysis for overall survival, local recurrence, and distant metastasis after propensity score-matching patients aged ≥80 years undergoing breast conservative surgery.

		All-Cause Death			Locoregional Recurrence			Distant Metastasis		
		aHR *	(95% CI)	*p* Value	aHR *	(95% CI)	*p* Value	aHR *	(95% CI)	*p* Value
Adjuvant WBRT	No	1		<0.0001	1		<0.0001	1		<0.0001
	Yes	0.56	(0.44–0.70)		0.29	(0.19–0.45)		0.45	(0.32–0.62)	
Age	80–84	1		<0.0001	1		0.6874	1		0.1827
	85–89	1.85	(1.28–2.69)		0.94	(0.61–1.45)		1.05	(0.71–1.56)	
	90+	1.67	(1.47–3.46)		0.77	(0.42–1.41)		0.66	(0.36–1.18)	
Differentiation	I	1		0.6671	1		0.3917	1		0.3724
	II	1.17	(0.90–1.88)		1.09	(0.76–1.71)		1.28	(0.79–1.40)	
	III	1.94	(0.97–2.19)		1.64	(0.77–2.75)		1.59	(0.69–2.46)	
AJCC clinical stage	I	1		0.4779	1		0.5677	1		0.3347
	II	1.08	(0.91–1.76)		1.18	(0.73–1.91)		1.14	(0.78–1.67)	
	III	1.12	(0.87–1.81)		1.75	(0.61–5.03)		1.59	(0.75–5.39)	
pT	pT0–1	1		0.7845	1		0.8537	1		0.7764
	pT2–4	1.06	(0.73–1.27)		1.05	(0.65–1.67)		1.06	(0.73–1.52)	
pN	pN0	1		0.0676	1		0.3442	1		0.3685
	pN1+	1.30	(0.98–1.73)		1.25	(0.79–2.00)		1.20	(0.81–1.77)	
Adjuvant chemotherapy	Yes	0.83	(0.43–1.12)	0.1168	0.93	(0.57–1.51)	0.7727	1.18	(0.78–1.80)	04319
Hormone receptor positive	Yes	0.88	(0.61–1.09)	0.2451	0.80	(0.55–1.18)	0.2617	0.84	(0.60–1.18)	0.3169
HER2 positive	Yes	1.06	(0.72–1.31)	0.2206	1.04	(0.75–1.18)	0.3494	1.14	(0.76–1.21)	0.4070
Nodal surgery	ALND	1		0.2361	1		0.2561	1		0.4612
	SLNB	0.77	(0.49–1.22)		1.16	(0.74–1.84)		1.05	(0.71–1.55)	
CCI Scores	0	1		0.4551	1		0.2721	1		0.0318
	1	1.09	(0.82–1.34)		0.90	(0.57–1.42)		0.94	(0.65–1.35)	
	2+	1.23	(0.81–1.79)		0.69	(0.43–1.09)		0.58	(0.38–0.89)	
Hospital level	Medical center	1		0.3925	1		0.1240	1		0.9823
	Non-Medical centers	1.11	(0.88–1.41)		0.73	(0.49–1.09)		1.00	(0.71–1.42)	

* All of the covariates listed in Table 2 were adjusted. WBRT—whole breast radiotherapy; aHR—adjusted hazard ratio; CI—confidence interval; AJCC—American Joint Committee on Cancer; HER2—human epidermal growth factor receptor 2; SLNB—sentinel lymph node biopsy; ALND—axillary lymph node dissection; CCI—Charlson comorbidity index; pT—pathologic tumor stage; pN—pathologic nodal stage.

**Table 3:** Multivariate analysis of overall survival of propensity score-matched patients undergoing breast conservative surgery, stratified by old (80 years or over) and very old (90 years or over).

		Age 80–89			Age ≥90		
		aHR *	(95% CI)	*p* Value	aHR *	(95% CI)	*p* Value
Adjuvant RT	No	1		0.0156	1		0.0040
	Yes	0.60	(0.40–0.91)		0.64	(0.48–0.87)	
Age	80–84	1		0.0382	–		
	85–89	1.48	(1.07–2.27)		–		
	90–94	–			1		0.0095
	95+	–			1.50	(1.10–2.04)	
Differentiation	I	1		0.2286	1		0.3581
	II	1.01	(0.58–1.76)		1.08	(0.84–1.94)	
	III	1.90	(0.93–2.50)		1.88	(0.90–2.32)	
AJCC clinical stage	I	1		0.4135	1		0.3453
	II	1.13	(0.88–1.54)		1.19	(0.83–1.70)	
	III	1.75	(0.78–2.02)		1.66	(0.70–2.48)	
pT	pT0–1	1		0.7816	1		0.8476
	pT2–4	1.07	(0.66–1.75)		1.04	(0.73–1.47)	
pN	pN0	1		0.1494	1		0.5985
	pN1+	1.36	(0.86–1.96)		1.11	(0.75–1.66)	
Adjuvant chemotherapy		0.64	(0.52–1.22)	0.2338	0.65	(0.52–1.91)	0.2533
HR positive		0.92	(0.60–1.39)	0.6880	0.92	(0.67–1.26)	0.6025
HER2 positive		1.12	(0.77–1.41)	0.5702	1.31	(0.87–1.96)	0.4925
Nodal surgery	ALND	1		0.8517	1		0.0102
	SLNB/+ALND	0.94	(0.57–1.57)		0.77	(0.52–1.15)	
CCI Scores	0	1		0.7365	1		0.8771
	1	1.11	(0.83–1.41)		1.07	(0.84–1.90)	
	2+	1.53	(0.86–2.10)		1.31	(0.85–2.42)	
Hospital level	Medical centers	1		0.8969	1		0.4276
	Non-medical centers	1.03	(0.68–1.57)		1.13	(0.84–1.52)	

* All of the covariates listed in Table 2 were adjusted. WBRT—whole breast radiotherapy; aHR—adjusted hazard ratio; CI—confidence interval; AJCC—American Joint Committee on Cancer; HER2—human epidermal growth factor receptor 2; SLNB—sentinel lymph node biopsy; ALND—axillary lymph node dissection; CCI—Charlson comorbidity index; RT—radiotherapy; pT—pathologic tumor stage; pN—pathologic nodal stage.

## Data Availability

The data sets supporting the study conclusions are included in this manuscript and its supplementary files.

## Supplementary Materials

The following are available online at https://www.mdpi.com/article/10.3390/jpm12020287/s1, Figure S1: Overall survival, LRR-free survival, and DM-free survival curves for propensity score matched patients aged 80–89 years receiving breast conservative surgery, Figure S2: Overall survival, LRR-free survival, DM-free survival curves for propensity score matched patients aged 90 years or over receiving breast conservative surgery.

## Abbreviations

WBRT, whole-breast radiotherapy; LRR, locoregional recurrence; DM, distant metastasis; IDC, invasive ductal carcinoma; BCS, breast-conserving surgery; OS, overall survival; aHR, adjusted hazard ratio; HR, hazard ratio; CI, confidence interval; AJCC, American Joint Committee on Cancer; TCRD, Taiwan Cancer Registry Database; SD, standard deviation; HER2, human epidermal growth factor receptor 2; SLNB, sentinel lymph node biopsy; ALND, axillary lymph node dissection; CCI, Charlson comorbidity index; ICD-10-CM, International Classification of Diseases, 10th Revision, Clinical Modification; NCCN, National Comprehensive Cancer Network; RT, radiotherapy; T, tumor; N, nodal; pT, pathologic tumor stage; pN, pathologic nodal stage; NHIRD, National Health Insurance Research Database; RCT, randomized controlled trial; PSM, propensity scores matching.
