# Supplementary material for: Adjuvant Radiotherapy Is Associated with an Increase in the Survival of Old (Aged over 80 Years) and Very Old (Aged over 90 Years) Women with Breast Cancer Receiving Breast-Conserving Surgery
Source: J Pers Med. 2022 Feb 16;12(2):287. doi: 10.3390/jpm12020287 (PMC8878030; doi:10.3390/jpm12020287)

Figure S1. Overall survival, LRR-free survival, and DM-free survival curves for propensity score matched patients aged 80-89 years receiving breast conservative surgery.

A. Overall survival curves

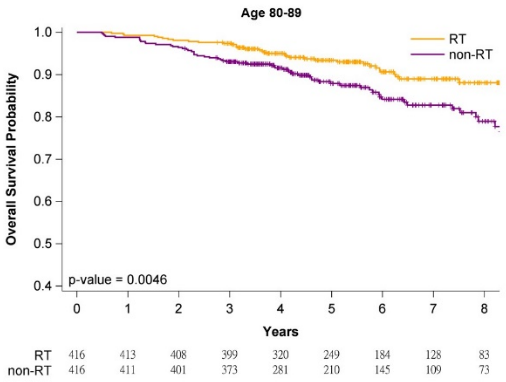

B. LRR-free survival curves

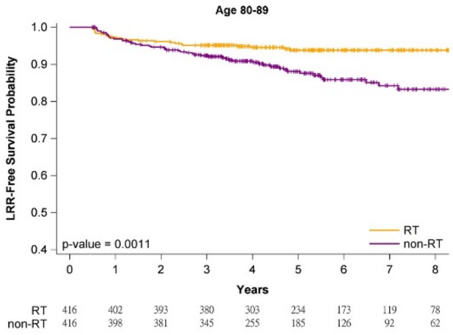

C. DM-free survival curves

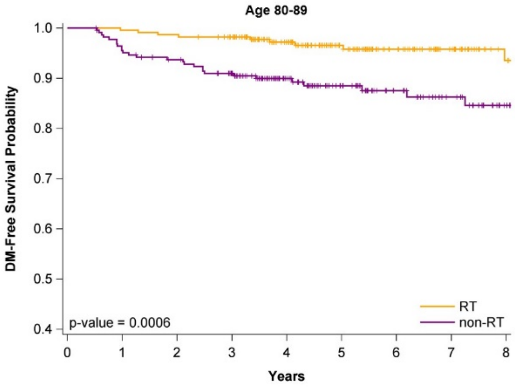

Figure S2. Overall survival, LRR-free survival, DM-free survival curves for propensity score matched patients aged 90 years or over receiving breast conservative surgery.

A. Overall survival curves

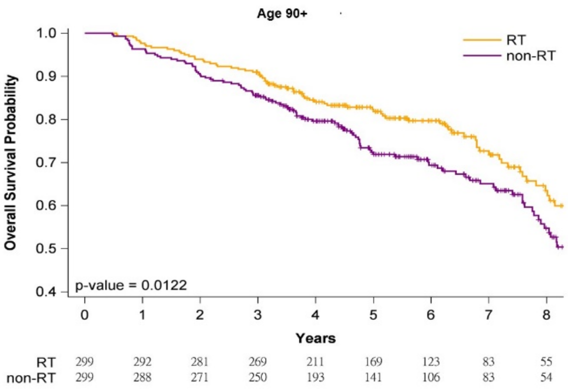

B. LRR-free survival curves

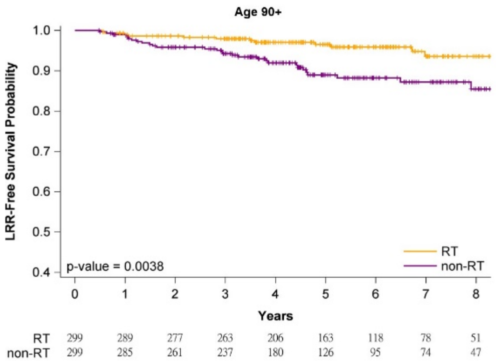

C. DM-free survival curves

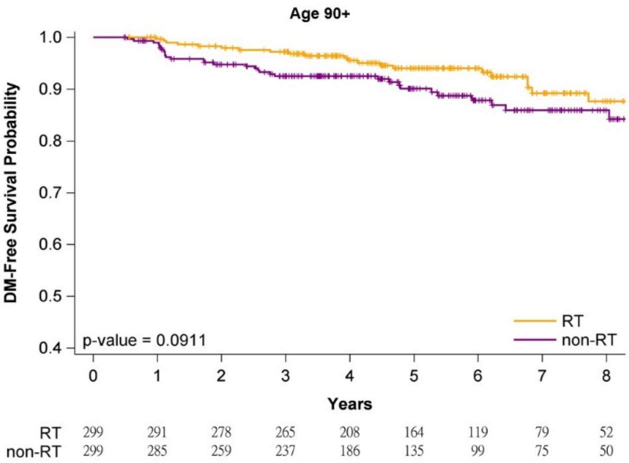

Supplement: Supplementary file 1 [file jpm-12-00287-s001.zip › jpm-1593800-supplementary.pdf]
